# Molecular Dynamics Simulation Study of the Interaction between Human Angiotensin Converting Enzyme 2 and Spike Protein Receptor Binding Domain of the SARS-CoV-2 B.1.617 Variant

**DOI:** 10.3390/biom11081244

**Published:** 2021-08-20

**Authors:** Priya Antony, Ranjit Vijayan

**Affiliations:** Department of Biology, College of Science, United Arab Emirates University, Al Ain P.O. Box 15551, United Arab Emirates; 201990021@uaeu.ac.ae

**Keywords:** SARS-CoV-2, spike protein, B.1.617 variant, delta variant, E484Q, L452R, molecular dynamics

## Abstract

The COVID-19 pandemic, caused by the SARS-CoV-2 virus, has had a significant impact on people’s daily lives. The rapidly spreading B.1.617 lineage harbors two key mutations—L452R and E484Q—in the receptor binding domain (RBD) of its spike (S) protein. To understand the impact and structural dynamics of the variations in the interface of S protein and its host factor, the human angiotensin-converting enzyme 2 (hACE2), triplicate 500 ns molecular dynamics simulations were performed using single (E484Q or L452R) and double (E484Q + L452R) mutant structures and compared to wild type simulations. Our results indicate that the E484Q mutation disrupts the conserved salt bridge formed between Lys31 of hACE2 and Glu484 of S protein. Additionally, E484Q, which could favor the up conformation of the RBD, may help in enhanced hACE2 binding and immune escape. L452R introduces a charged patch near the binding surface that permits increased electrostatic attraction between the proteins. An improved network of intramolecular interactions observed is likely to increase the stability of the S protein and conformational changes may prevent the binding of neutralizing antibodies. The results obtained from the molecular dynamics simulations suggest that structural and dynamic changes introduced by these variations enhance the affinity of the viral S protein to hACE2 and could form the basis for further studies.

## 1. Introduction

The coronavirus disease 2019 (COVID-19) pandemic, caused by the severe acute respiratory syndrome coronavirus 2 (SARS-CoV-2), has had a catastrophic impact worldwide. Since its emergence in December 2019, the total number of confirmed cases has exceeded 200 million and it has caused over 4 million deaths. The year 2020 was a challenging one, and one of the highlights was the expedited development of several COVID-19 vaccines with impressive efficacy [1]. However, strategies to contain the pandemic have been affected by the emergence of new viral variants possessing increased transmissibility and/or capability to escape the immune system.

**Figure 1:**
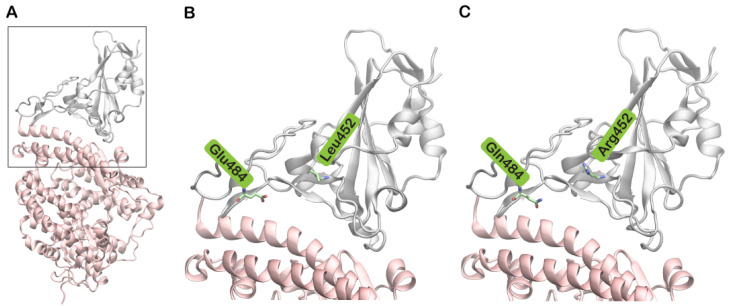
Structures of SARS-CoV-2 spike (S) protein receptor-binding domain (RBD) bound to human ACE2 (hACE2). (**A**) SARS-CoV-2 S protein RBD (grey) bound to hACE2 (pink) based on Protein Data Bank (PDB) structure with PDB ID: 6M0J; (**B**) The boxed region in A is enlarged showing the wild type residues Leu452 and Glu484 in stick representation. (**C**) The boxed region in A is enlarged showing the mutated residues Arg452 and Gln484.

SARS-CoV-2 is an RNA virus and its genome encodes 16 non-structural (nsp1–16) and four structural proteins, namely spike (S), nucleocapsid (N), membrane (M), and envelope (E). The S protein, a type 1 fusion protein, is primarily involved in the invasion of the host cell by SARS-CoV-2 [2]. Several studies have reported that the human angiotensin-converting enzyme (hACE2) serves as a high-affinity receptor for the receptor-binding domain (RBD) of the S protein (Figure 1A) [3]. 

Like other viruses, SARS-CoV-2 mutates and evolves as it replicates. A large number of SARS-CoV-2 genomes have now been sequenced. This provides impressive insights into viral variants and their spread over time. These variations are routinely monitored by sequencing studies, epidemiological investigations and molecular studies. Even though thousands of variants emerged in the early phase of the infection, most of these did not have a significant impact on viral spread and infectivity [2]. However, in April 2020, the original SARS-CoV-2 virus acquired the D614G mutation in the S protein [4]. The now ubiquitous D614G variant exhibited higher transmissibility and infectivity with efficient replication [5]. As the pandemic rages on, multiple genomic variants of SARS-CoV-2 have emerged in different parts of the world including the United Kingdom (UK), South Africa, Brazil, and India. The dramatic rise in COVID-19 cases in the UK has been attributed to a variant named B.1.1.7 or 20I/501Y.V1. Sequencing analysis identified several mutations in the spike region including D614G. Of these, N501Y appeared to be the major mutation in the RBD of the S protein [6]. Epidemiological studies indicated that this variant has up to 80% higher transmissibility and was associated with an increased risk of death when compared to previously reported variants [7]. The South African variant B.1.351 or 20H/501Y is characterized by three mutations in the RBD region of the S protein—K417N, E484K, and N501Y [8]. Both variants (B.1.351 and B.1.1.7) share the N501Y mutation that aids in increased binding affinity and immune escape [9]. The variant P.1, originally reported in Brazil, harbors N501Y, E484K, K417T mutations in the S protein. These variants were classified as ‘variants of concern’ (VOCs) due to their higher transmissibility, immune escape capability, lower response to vaccines and severity of the disease. To disassociate variants and country names, a new naming system was recently devised by the World Health Organization (WHO). Under this naming convention, the B.1.1.7 variant, originally identified in the UK, was called alpha, the B.1.351 variant was named beta, and the P.1 variant as gamma [10]. 

Since early March 2021, the numbers of reported COVID-19 cases and deaths have risen quite sharply in India. Phylogenetic analysis revealed that the major viral variant circulating in India belonged to the newly identified lineage B.1.617. This lineage includes three main subtypes (B1.617.1, B.1.617.2, and B.1.617.3) harboring several mutations in the S protein, including the synonymous D111D variation and the nonsynonymous G142D, L452R, E484Q, D614G and P681R variations. Of these, the combination of E484Q and L452R is of particular concern as they are positioned in the receptor-binding motif (RBM) of the S protein (Figure 1B,C). Even though crystal structures of both RBDs of SARS-CoV-2 and SARS-CoV share the same scaffold, sequence differences in the RBM region of SARS-CoV-2 promote greater electrostatic complementarity with hACE2. This in turn provides enhanced binding affinity of SARS-CoV-2 to hACE2 compared to SARS-CoV [11]. The combination of these two mutations, along with other mutations, gives the virus survival advantage and the ability to spread rapidly. The B.1.617 lineage has received particular attention due to its increased infectivity, high virulence capacity, and potential immune escape. Currently, while vaccination programs have been implemented in several nations to a limited extent, this wave of COVID-19 infections is creating serious global health concerns.

Gaining a deeper understanding of the interactions between the viral S protein and hACE2 is crucial for developing drugs and vaccinations as well as for ascertaining the efficacy of existing vaccinations against novel SARS-CoV-2 variants. Our previous study evaluated the dynamic interactions of SARS-CoV-2/SARS-CoV RBD with hACE2 and identified important interactions that assist the stable binding of SARS-CoV-2 when compared to SARS-CoV [12]. Here, we extend this to investigate the structural stability, binding affinity and intermolecular polar and hydrophobic contacts in the S protein RBD of the B.1.617 variant—E484Q and L452R independently and in combination (E484Q + L452R)—using multiple 500 ns molecular dynamics (MD) simulations and binding free energy calculations.

## 2. Materials and Methods

Coordinates of the three-dimensional X-ray crystal structures of the SARS-CoV-2 RBD bound to hACE2 were obtained from the Protein Data Bank (PDB; PDB ID: 6M0J). Single and double mutant complexes were created by mutating the amino acids Glu484 to Gln484 and Leu452 to Arg452 using Schrödinger Maestro 2019-4 (Schrödinger, LLC, New York, NY, USA). These mutant complexes were first pre-processed using the Protein Preparation Wizard of Schrödinger (Schrödinger, LLC, New York, NY, USA). The protein preparation process involved assigning the correct bond order, creating disulfide bonds, adjusting ionization states, removing unwanted water molecules, metals and cofactors, correcting the orientation of groups, capping the termini, adding missing atoms and sidechains and assigning partial charges. Hydrogen atoms were incorporated, and a standard protonation state at pH 7 was used. The structures of the single and double mutant spike protein RBD bound to ACE2 were placed in orthorhombic boxes of size 125 Å × 125 Å × 125 Å and solvated with single point charge (SPC) water molecules using the Desmond System Builder (Schrödinger, LLC, New York, NY, USA). All simulation systems were neutralized with counter ions and a salt concentration of 0.15 M NaCl was maintained. The simulations were performed for 500 ns using Desmond [13] in triplicate with different set of initial velocities assigned to each atom. The systems were described using the OPLS forcefield. Before the production run, all simulation systems were subjected to Desmond’s default eight-stage relaxation protocol. The isotropic Martyna–Tobias–Klein barostat and the Nose–Hoover thermostat were used to maintain the pressure at 1 atm and temperature at 300 K, respectively [14,15]. Long-range coulombic interactions were evaluated using the smooth particle mesh Ewald method and the short-range cutoff was set as 9.0 Å [16]. A time-reversible reference system propagator algorithm (RESPA) integrator was employed with an inner time step of 2.0 fs and an outer time step of 6.0 fs [17]. The binding free energy of all three mutant complexes was evaluated using the molecular mechanics generalized Born surface area (MM-GBSA) approach. Frames were extracted every 10 ns from MD simulation trajectories and MM-GBSA based binding free energy was computed using Schrödinger Prime employing the VSGB 2.0 solvation model [18]. The data obtained from the simulations were analyzed using packaged and in-house scripts. MD trajectories were analyzed to identify critical interactions that were formed, retained and disrupted in the interface between S-RBD and hACE2. Structural stability of the single mutant and double mutant complexes were examined using root mean square deviation (RMSD), root mean square fluctuation (RMSF) and radius of gyration (Rg). Surface potential of the wild type and L452R mutants was calculated using the Adaptive Poisson–Boltzmann Solver (APBS) in Schrödinger Maestro. Graphs were plotted using R version 3.6.3 (https://www.r-project.org, accessed on 8 July 2020) and images of structures were generated using Visual Molecular Dynamics version 1.9.3 [19].

## 3. Results and Discussion

**Figure 2:**
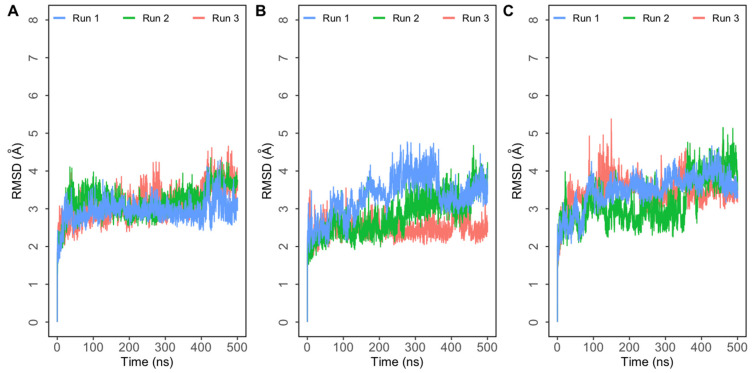
Root mean square deviation (RMSD) of protein backbone atoms with respect to the initial structure obtained from three independent 500 ns simulations of SARS-CoV-2 spike (S) protein bound to human ACE2 (hACE2). (**A**) Simulations of double mutant E484Q + L452R complex; (**B**) simulations of E484Q complex; (**C**) simulations of L452R complex.

Molecular dynamics simulations trajectories of three systems—single mutants E484Q and L452R, and double mutant E484Q + L452R—in triplicate were analyzed. RMSD plots of the three complexes indicated that these systems reached equilibrium quickly and the RMSD of the complexes stabilized around 4 Å in all simulations (Figure 2).

**Figure 3:**
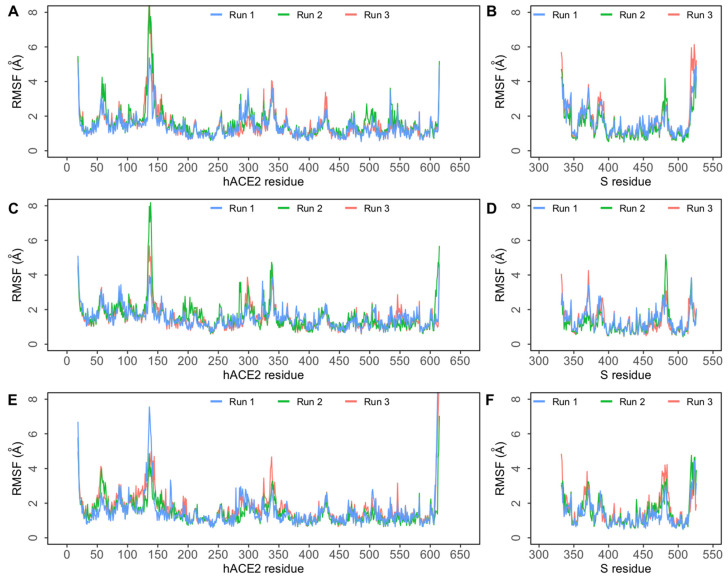
Root mean square fluctuation (RMSF) of protein Cα atoms obtained from three independent 500 ns simulations of SARS-CoV-2 spike (S) protein bound to human ACE2 (hACE2). (**A**) RMSF of Cα atoms of hACE2 protein in the E484Q + L452R complex; (**B**) RMSF of Cα atoms of SARS-CoV-2 S RBD in the E484Q + L452R complex; (**C**) RMSF of Cα atoms of hACE2 protein in the E484Q complex; (**D**) RMSF of Cα atoms of SARS-CoV-2 S RBD in the E484Q complex; (**E**) RMSF of Cα atoms of hACE2 protein in the L452R complex; (**F**) RMSF of Cα atoms of SARS-CoV-2 S RBD in the L452R complex.

To assess residue-level protein fluctuations and backbone flexibility RMSF of backbone Cα atoms were computed and plotted (Figure 3). This is particularly relevant for residues in the S-hACE2 interface. The binding interface of S RBD consists of four loop regions—loop 1: residues 438–450, loop 2: residues 455–470, loop 3: residues 471–491, and loop 4: residues 495–508. These provide the necessary flexibility while binding to hACE2. Studies have suggested that the loop 3 and loop 4 regions are the most flexible regions in the RBD [20]. Closer observation of the RMSF plot (Figure 3) revealed that in all three complexes, higher flexibility was observed in the loop region between 470–490. In all three complexes, the fluctuation of the ACE2 backbone was comparable. As indicated by the flat Rg plots (Supplementary Figure S1), the overall compactness and secondary structure elements of the protein complexes were found to be preserved throughout the simulation.

**Figure 4:**
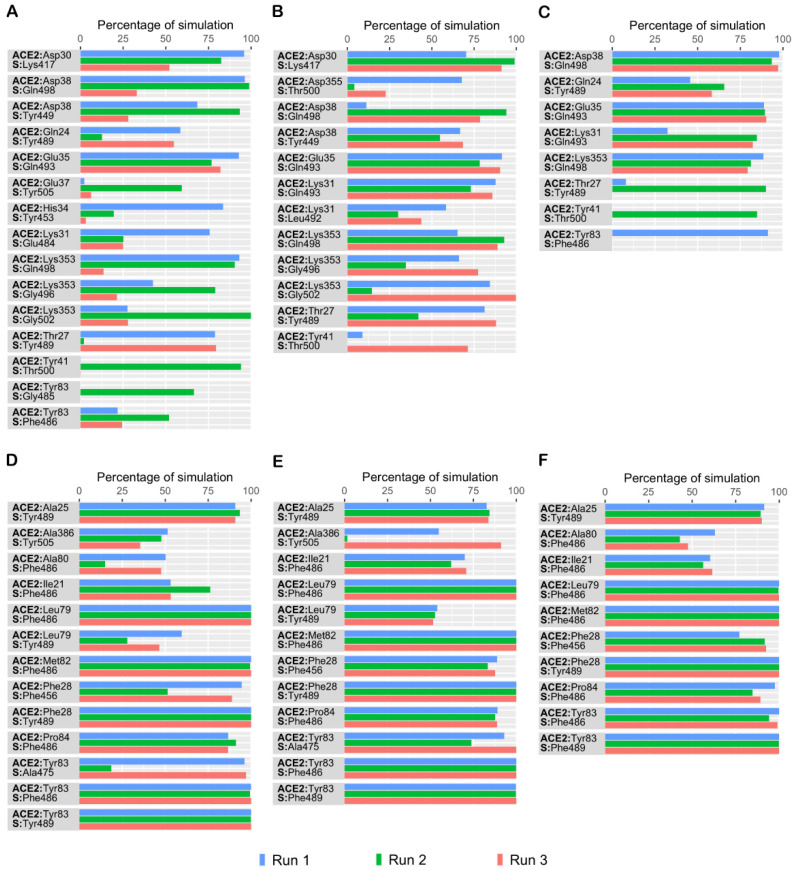
The percentage of simulation time during which intermolecular contacts were retained between human ACE2 (hACE2) and SARS-CoV-2 spike (S) protein receptor-binding domain (RBD) residues. (**A**) Intermolecular polar contacts in the L452R mutant complex; (**B**) Intermolecular polar contacts in the E484Q mutant complex; (**C**) Intermolecular polar contacts in the double mutant E484Q + L452R complex; (**D**) Intermolecular hydrophobic contacts in the L452R mutant complex; (**E**) Intermolecular hydrophobic contacts in the E484Q mutant complex; (**F**) Intermolecular hydrophobic contacts in the double mutant E484Q + L452R complex.

Throughout the simulation, several intermolecular polar and hydrophobic contacts were observed to form, break and reform in the protein complexes. To elucidate how the single mutant and double mutant affects the interaction pattern of SARS-CoV-2 RBD and hACE2 interaction at the molecular level, these data were compared to our previously reported wildtype data [12]. Residue level interactions sustained for at least 50% of simulation time in at least one simulation are presented in Figure 4. The data revealed that, in both single mutant complexes, intermolecular interactions observed were similar to wildtype data. Four interfacial residues of spike protein—Lys417, Gln493, Tyr449, and Gln498—in the single mutant complexes interacted with Asp30, Glu35, Asp38 and Lys353 of hACE2, as reported previously [12]. Apart from these conserved interactions, in the E484Q mutant complex, the backbone and sidechain of Lys353 in hACE2 interacted with Gly502 and Gly496 of S RBD (Figure 4B). Additionally, the sidechain of Lys31 of hACE2 consistently interacted with Gln493 of S protein in all three simulations, while the sidechain of Gln498 in S protein also showed consistent interaction with Asp38 of hACE2. Simulation data revealed that in both single mutant complexes Thr27 of hACE2 interacted with Tyr489 of S protein. Besides these interactions, residues of single mutant L452R, Tyr489 and Gly496 of S protein, interacted with Gln24 and Lys353 of hACE2, respectively (Figure 4A). Normally, Lys417, on the S-RBD surface, forms a salt bridge with Asp30 of hACE2. Unlike the single mutant complexes, this conserved interaction was notably absent in the double mutant complex (Figure 4C). Additionally, in both single mutants, Asp38 of hACE2 interacted with Tyr449 of the S protein. However, in the double mutant complex Asp38 was observed to interact with Gln498 of S protein (Figure 4C). Similar to the E484Q, in the double mutant complex, the sidechain of Lys31 of hACE2 consistently interacted with Gln493 of the S protein in all simulations (Figure 4C).

To gain a deeper understanding of the effect of the mutated residues, the interaction pattern of the three complexes was evaluated and compared to the wild type simulation. Glu484 (E484), situated on a flexible loop of spike RBD, forms a salt-bridge with Lys31 of hACE2. Lys31 and Lys353 on the surface of hACE2 are regarded as virus binding hotspots [21]. In the double mutant, residues Lys31 and Glu35 of hACE2 consistently interacted with Gln493, while Asp38 and Lys353 formed hydrogen bonds with Gln498 of the spike protein (Figure 4C). The formation of a salt bridge between Lys31 of hACE2 and Glu484 of S enhances the affinity of SARS-CoV-2 to hACE2 when compared to SARS-CoV [22]. After Glu484 was mutated to Gln484, this salt bridge was disrupted in the E484Q and double mutant complexes. However, as expected, this salt bridge was observed in the L452R single mutant complex (Figure 4A). Hence, the simulation data suggests that the E484Q mutation could play a role in disrupting the interfacial interaction. However, studies indicate an increased fitness of the E484Q mutant when compared to the wild type [23]. This paradox may be explained by considering the dynamic state of the RBD. The RBD of SARS-CoV-2 exhibits two conformational states—a down/closed conformation, in which the receptor binding region is not exposed, and an up/open state that allows receptor binding [24]. Cryo-EM studies have suggested that the RBD of SARS-CoV-2 exists mostly in the down state [25]. Apart from forming the intermolecular salt bridge, Glu484 also stabilizes the RBD down conformation by forming an intramolecular hydrogen bond with Phe490 [26]. In the present analysis, Glu484 showed strong interactions with Phe490, while the mutant Gln484 formed fewer interactions with Phe490. Since this interaction is weaker in the E484Q mutant complex, it could favor the up conformation of the RBD and result in higher hACE2 binding affinity and immune escape. In agreement with this, Gobeil et al. reported that the E484K mutation causes conformational changes in the RBD that favors the up state and reduces antibody binding [26]. Thus, conformational changes in the RBD, induced by the E484Q mutation, could impact the stability of the hACE2 binding surface. Furthermore, Glu484 is as an important antibody escape site of SARS-CoV-2 and mutations at this location could impact the binding and neutralization by antibodies [27]. Studies have also demonstrated that the E484Q mutation could reduce the neutralization by plasma and antibodies by 10-fold [28]. This variant is also resistant to bamlanivimab, a recombinant antibody approved for the COVID-19 treatment, by failing to block viral entry mediated by glycoproteins [29].

**Figure 5:**
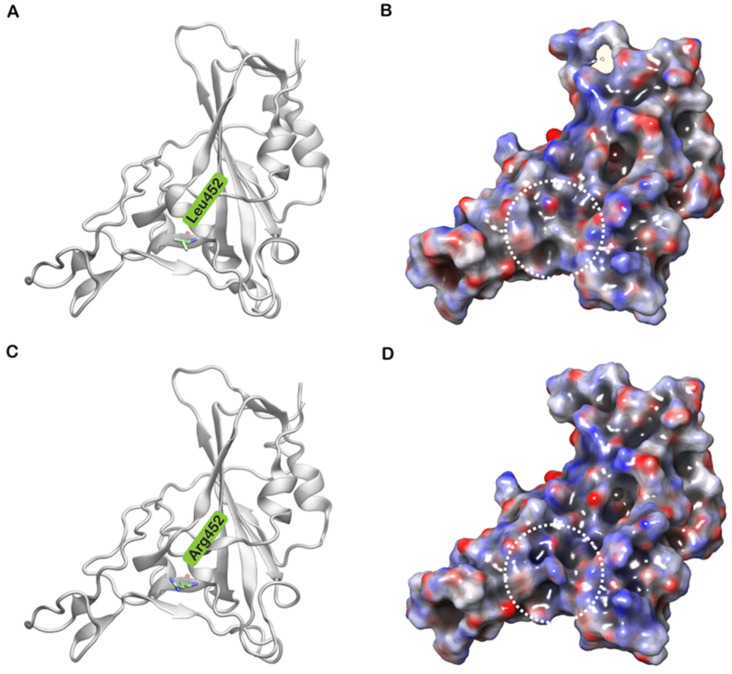
(**A**) Cartoon representation of the SARS-CoV-2 spike (S) protein receptor-binding domain (RBD) of wild type with Leu452 shown in stick representation. (**B**) The electrostatic potential, calculated using Adaptive Poisson–Boltzmann Solver (APBS), mapped on to the surface of the wild type protein. (**C**) Cartoon representation of the S protein RBD of the mutated Arg452 shown in stick representation. (**D**) The electrostatic potential, calculated using APBS, mapped on to the surface of the mutant protein (L452R).

It has been reported that Leu452, in spite of being located in the RBM region, does not directly interact with hACE2 [30]. However, Leu452 (Figure 5A), together with Phe490 and Leu492, forms a hydrophobic patch on the surface of the S protein (Figure 5B) [31]. A mutation to a highly polar and hydrophilic arginine (Figure 5C) could potentially introduce local perturbations (Figure 5D) that could affect how it interacts with a complementary surface. Additionally, Leu452 is a hotspot located in close proximity to the negatively charged residues Glu35, Glu27 and Asp38 of hACE2 [32]. The incorporation of additional charged residues in the vicinity of the binding interface could increase the electrostatic attraction between two proteins. Hence, the mutation of leucine to a positively charged arginine enhances electrostatic complementarity in the interface (Figure 5D). Compared to Leu452, Arg452 was observed to interact more with nearby residues including Ser349, Tyr351, Phe490, Leu492 and Ser494. The increased intramolecular interactions could thus increase the stability of the S protein. The role of this mutation in neutralizing antibodies and sera from convalescent patients has been investigated [28]. The changes induced by L452R mutation prevents the binding of neutralizing antibodies and promotes high infectivity.

The stability of hydrogen bond interactions in the single and double mutant complexes was also monitored (Supplementary Figure S2). The number of intermolecular hydrogen bonds is also higher in the mutant complexes (mean ± SD for E484Q+L452R simulations: 10.8 ± 2.1, 10.4 ± 1.9, 10.3 ± 2.0; E484Q simulations: 11.4 ± 2.2, 11.6 ± 1.9, 14.4 ± 2.1; L452R simulations: 11.9 ± 2.0, 13 ± 1.9, 8.9 ± 2.2).

In support of our results, Cherian and colleagues reported that the L452R mutation, and E484Q, could reduce intramolecular and intermolecular interactions and disrupts an electrostatic bond with Lys31 of hACE2 [27]. A change from a hydrophobic leucine on the protein surface to arginine (L452R) also increases its interactions with water molecules that could further stabilize the protein. The double mutant complex, harboring a combination of these two mutations, is capable of escaping neutralizing antibodies, increasing viral infectivity and viral replication.

Residues that were involved in hydrophobic interactions in the S-hACE2 interface are included in Figure 4. All three complexes formed consistent hydrophobic contacts with hACE2 but when compared to the double mutant, single mutants exhibit slightly higher numbers of hydrophobic contacts. In all three protein complexes, Ile21, Leu79, Met82, Tyr83, and Pro84 of hACE2 exhibited consistent contact with Phe486 of the S protein whereas Tyr489 interacted with Ala25, Phe28 and Tyr83 of hACE2 in all three runs. Compared to the wild type data, additional hydrophobic contacts involving Ile21, Ala25, and Pro84 of hACE2 were observed in the mutant complexes (Figure 4D–F). All three protein mutant complexes were observed to form similar hydrophobic interactions in the simulations. Phe28 of hACE2 was observed to form sustained contacts with Phe456 in all three complexes. In both single mutant complexes, Tyr83 formed hydrophobic contacts with Ala475 of hACE2 (Figure 4D,E). This interaction was not observed in any of the double mutant simulations.

To compare how mutations in S RBD affect the binding free energy (ΔG_bind_), it was estimated, using frames extracted from all MD simulations, based on the MM-GBSA approach. The binding free energy of the double mutant simulations were −147.55 ± 16.22 kcal/mol, −132.83 ± 18.71 kcal/mol, and −127.80 ± 21.30 kcal/mol, respectively. In the single mutant complex involving the E484Q mutant, ΔG_bind_ values computed were −131.25 ± 20.95 kcal/mol, −143.47 ± 14.96 kcal/mol, and −149.14 ± 20.98 kcal/mol, and for the L452R mutant the values were −146.58 ± 19.70 kcal/mol, −148.60 ± 17.67 kcal/mol, and −117.22 ± 22.15 kcal/mol. Importantly, compared to the wild type, all the mutant complexes exhibited a more favorable ΔG_bind_. This supports the higher binding affinity of the mutants compared to the wild type virus [33].

In summary, this study looks at the structural dynamics of the S protein-hACE2 interface of the rapidly spreading B.1.617 variant using multiple molecular dynamics simulations. Single and double mutant simulations were used to identify the differences in interactions and structural conformations of the spike protein when compared to the wild type. Overall, the data provide deeper insights into the higher affinity of the B.1.617 variant of SARS-CoV-2 and could form the basis for further studies.

## 4. Conclusions

Even though a conserved salt bridge is absent, the E484Q mutation could favor the open conformation of RBD that aids in enhanced hACE2 binding and immune escape. The L452R mutation enhances the electrostatics of the binding surface and aids electrostatic attraction between hACE2 and the S protein. The enhanced network of intramolecular interactions increases the stability of the S protein and the conformational changes induced by these mutations could prevent the binding of neutralizing antibodies and promote higher infectivity. The results presented here are based on molecular dynamics simulations. Further, wet-lab studies are essential to fully validate these observations.

## Supplementary Materials

The following are available online at https://www.mdpi.com/article/10.3390/biom11081244/s1, Figure S1: Radius of gyration (Rg) of human ACE2 (hACE2) and spike (S) protein of SARS-CoV-2 receptor-binding domain (RBD) from three 500 ns simulations, Figure S2: Hydrogen bonds between human ACE2 (hACE2) and spike (S) protein of SARS-CoV-2 receptor-binding domain (RBD) from three 500 ns simulations.
