# Supplementary material for: Molecular Dynamics Simulation Study of the Interaction between Human Angiotensin Converting Enzyme 2 and Spike Protein Receptor Binding Domain of the SARS-CoV-2 B.1.617 Variant
Source: Biomolecules. 2021 Aug 20;11(8):1244. doi: 10.3390/biom11081244 (PMC8391770; doi:10.3390/biom11081244)
Supplement: Supplementary file 1 [file biomolecules-11-01244-s001.zip › biomolecules-1324610-supplementary.pdf]

Molecular dynamics simulation study of the interaction between human angiotensin converting enzyme 2 and spike protein receptor binding domain of the SARS-CoV-2 B.1.617 variant

Priya Antony[1] and Ranjit Vijayan[1,*]

[1] Department of Biology, College of Science, United Arab Emirates University, PO Box 15551, Al Ain, United Arab Emirates

[*] Correspondence: ranjit.v@uaeu.ac.ae

SUPPLEMENTARY MATERIALS

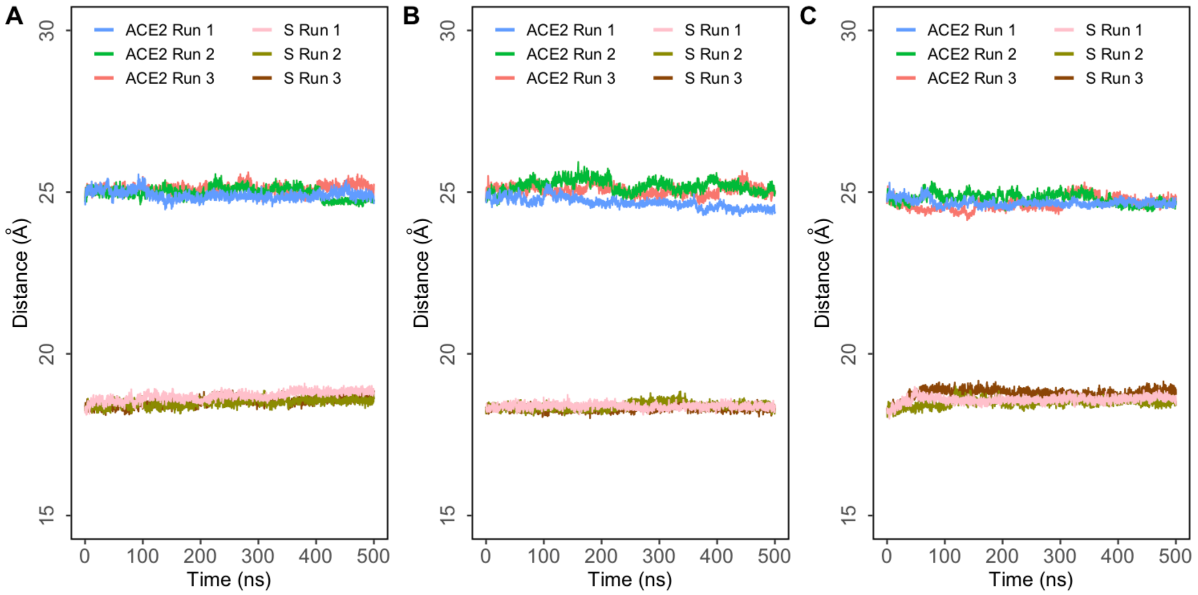

Figure S1. Radius of gyration (Rg) of human ACE2 (hACE2) and spike (S) protein of SARS-CoV-2 receptor-binding domain (RBD) from three 500 ns simulations of **(A)** E484Q+L452R; **(B)** E484Q; **(C)** L452R.

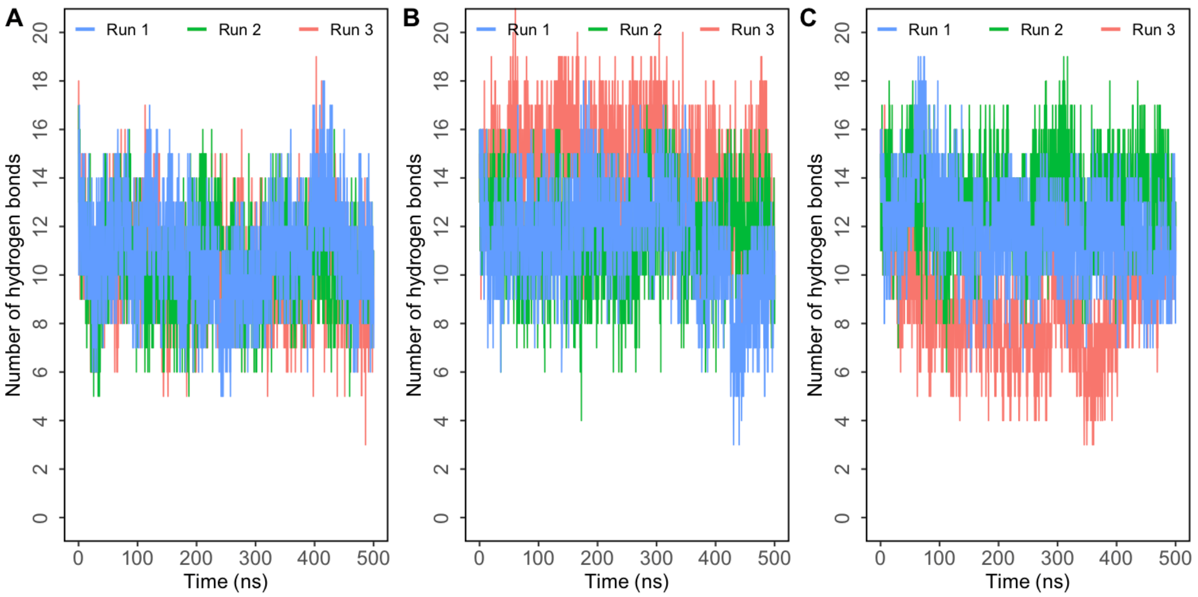

Figure S2. Hydrogen bonds between of human ACE2 (hACE2) and spike (S) protein of SARS-CoV-2 receptor-binding domain (RBD) from three 500 ns simulations of **(A)** E484Q+L452R; **(B)** E484Q; **(C)** L452R.
